# Efficacy of individualized orelabrutinib-based regimens in relapsed or refractory central nervous system lymphoma

**DOI:** 10.3389/fneur.2025.1570224

**Published:** 2025-06-05

**Authors:** Yuchen Wu, Xuefei Sun, Liwei Lv, Qu Cui, Jun Qian, Ruixian Xing, Xueyan Bai, Yuedan Chen, Qing Liu, Wenyuan Lai, Chunji Gao, Shengjun Sun, Nan Ji, Yuanbo Liu

**Affiliations:** ^1^Department of Hematology, Beijing Tiantan Hospital, Capital Medical University, Beijing, China; ^2^Department of Hematology, Beijing Tongren Hospital, Capital Medical University, Beijing, China; ^3^Department of Hematology, Chinese PLA General Hospital, Beijing, China; ^4^Neuroimaging Center, Beijing Tiantan Hospital, Capital Medical University, Beijing, China; ^5^Department of Neurosurgery, Beijing Tiantan Hospital, Capital Medical University, Beijing, China

**Keywords:** central nervous system lymphoma, relapsed or refractory, Bruton’s tyrosine kinase inhibitor, orelabrutinib, methotrexate

## Abstract

**Background:**

Relapsed or refractory central nervous system lymphoma (rrCNSL) lacks established preferred treatment and carries an inferior prognosis. Bruton’s tyrosine kinase inhibitor (BTKi) showed promising effectiveness. Orelabrutinib is a second-generation BTKi with a high concentration in cerebrospinal fluid.

**Methods:**

In this retrospective analysis, the outcomes of 37 relapsed or refractory central nervous system diffuse large B-cell lymphoma patients who received orelabrutinib, high-dose methotrexate, ifosfamide, etoposide, and dexamethasone (Ore-MIED) or orelabrutinib, high-dose methotrexate, temozolomide and dexamethasone (Ore-MTD) were evaluated.

**Results:**

Of the 37 patients included, 11 received Ore-MTD, and 26 received the Ore-MIED regimen. The overall response rate in our cohort was 89.2%, with complete remission achieved in 51.4% of patients and partial remission in 37.8% of patients. The median progression-free survival was observed to be 7.0 months. No statistically significant difference was found in the median progression-free survival between patients receiving different treatment regimens (5.0 months for Ore-MTD versus 13.0 months for Ore-MIED; *p* = 0.29). Moreover, the median overall survival has not been reached in this cohort, indicating a promising outcome despite the aggressive nature of the disease.

**Conclusion:**

Our study confirms the effectiveness and safety of Ore-MIED/Ore-MTD in rrCNSL patients, even in those with previous exposure to multiple lines of treatment.

## Introduction

Central nervous system lymphoma encompasses primary central nervous system lymphoma (PCNSL) and secondary central nervous system lymphoma (SCNSL). It is characterized by its aggressive nature and associated with a dismal prognosis. Despite recent progress and improved survival in PCNSL, relapses remain frequent, especially within the first 2 years after initial response (1–3). Additionally, approximately one-third of newly diagnosed PCNSL patients were primary resistant to first-line treatment (4). To date, no consensus exists on optimal management strategies for relapsed or refractory (rrCNSL), underscoring a critical unmet clinical need (5). Salvage therapies include high-dose methotrexate (HD-MTX) rechallenge (2), temozolomide (6), whole brain radiotherapy (WBRT) (7), lenalidomide (8), thiotepa/rituximab/ifosfamide/etoposide (TIER), intensive chemotherapy followed by autologous hematopoietic stem cell transplantation (ASCT) (9, 10). These studies show heterogeneous response rates (ranging from 31–67%) and survival times (ranging from 2.8–50 months).

Consistent with the crucial role of aberrant B-cell receptor signaling pathways in PCNSL, Bruton tyrosine kinase inhibitors (BTKis) have dramatically improved the treatment efficacy in rrPCNSL (11). The combination of rituximab, HD-MTX and ibrutinib led to an overall response rate (ORR) of 80% with a median progression-free survival (PFS) of 9.2 months in rrCNSL in a phase 1b study (12, 13).

Orelabrutinib is a second-generation irreversible BTKi that exhibits a high ability to penetrate the blood–brain barrier, which results in a high cerebrospinal fluid (CSF)/plasma ratio. Herein, we explore the feasibility and efficacy of a combined regimen including orelabrutinib, HD-MTX, ifosfamide, etoposide and dexamethasone (Ore-MIED) in comparison with orelabrutinib, HD-MTX, temozolomide and dexamethasone (Ore-MTD) in a single series of patients with rrCNSL.

## Methods

### Patients

We conducted a retrospective analysis of 37 consecutive patients with relapsed or refractory central nervous system lymphoma (rrCNSL) treated at the hematology department of Beijing Tiantan Hospital from March 1st, 2021, to December 20th, 2022. The inclusion criteria were as follows: (1) a histological confirmation of central nervous system diffuse large B-cell lymphoma (DLBCL) with or without systemic disease; (2) relapsed or refractory disease; (3) received orelabrutinib-based regimens. Relapse (Rel) disease refers to recurrence after the CR of initial chemotherapy. Refractory (Ref) status was determined based on the fulfillment of any of the following criteria: inadequate tumor shrinkage (<50%) or disease progression observed after four courses of chemotherapy; achieving complete remission (CR) with standard chemotherapy followed by relapse within 6 months; experiencing two or more relapses after achieving CR; or relapse occurring after autologous stem cell transplantation (ASCT). Each participant in this study provided written consent after being informed and fully aware of the study details.

The research protocol for this study was approved by the Ethics Committee of Beijing Tiantan Hospital, Capital Medical University (Ethical approval reference number: KY 2020–066-02). Written informed consent was obtained from patients upon admission to the Department of Neurosurgery or the Department of Hematology, prior to the initiation of chemotherapy.

### Treatment

All patients received orelabrutinib-containing chemotherapy. The regimen option was made by physicians concerning the patients’ previous therapy and the quality of response to prior therapy and the duration of remission. Regimens include Ore-MTD and Ore-MIED (regarding drug dose, see Supplementary Data 1). Orelabrutinib was administered in line with chemotherapy continuously orally 150 mg per day. Orelabrutinib combined with chemotherapy was administered every 3 weeks for 3–6 cycles.

### Assessment

Enhanced MRI was performed every cycle, and CSF examination and ocular slit lamp were performed every three cycles. Patients with ocular involvement were examined by an ophthalmologist every cycle. In addition, whole-body CT with a positron emission tomography scan (PET-CT) was required in SCNSL. Treatment response of CNS disease was assessed using the International Primary CNS Lymphoma Collaborative Group (IPCG) Evaluation and Response Criteria. The response of systemic lymphoma was graded according to the 2014 Lugano criteria. The composite of overall objective response rate (ORR) was determined by the combining rates of complete response (CR), unconfirmed complete response (CRu), and partial response (PR) rates. PFS was assessed from the time of initiation of relapsed or refractory disease treatment to the time of disease progression or death. Overall survival (OS) was defined as time from the initiation of relapsed or refractory disease treatment to death from any cause. Toxicities were assessed and recorded using the Common Terminology Criteria for Adverse Events Version 5.0.

### Statistical analysis

Statistical analyses were performed using SPSS 29.0 and GraphPad Prism version 9.0. All tests were two-sided, and a *p*-value < 0.05 was considered statistically significant. The univariate analysis for PFS was conducted using Cox proportional hazards regression models, multivariate analysis was selected based on their significance in univariate Cox regression (*p* < 0.1).

## Results

### Patient characteristics

We included 34 (91.9%) rrPCNSL patients and 3 (8.1%) rrSCNSL patients in this retrospective study (Table 1). There were 22 males and 15 females with a median age of 55 (range 33–83 years). Twenty-four (64.9%) of the patients were non-GCB, 3 (8.1%) were GCB using Hans algorithm, and 10 (27.0%) were unknown. Eighteen (48.6%) were detected to have BCL-2 and c-myc double expression. At the initial disease, 27 (73%) patients had disease involving deep brain lesions (basal ganglia, periventricular tissue, brainstem, cerebellum). The median IELSG score was 3 (range 0–5).

**Table 1:** Baseline characteristics of included patients.

Characteristics	*N* = 37
Gender (M/F, *n*/%)	22 (59.5)/15 (40.5)
Age (years, median, range)	(55, 33–83)
≤60	27(73.0)
>60	10(27.0)
CNSL (*n*, %)	
PCNSL	34(91.9)
SCNSL	3(8.1)
Subtype of DLBCL	
Non-GCB	24(64.9)
GCB	3(8.1)
Unknown	10(27.0)
IELSG score (median, range)	3(0–5)
Involved of deep lesions	27(73.0)
Bcl-2 and c-myc double expression	18(48.6)
Type of disease	
Relapse	11(29.7)
Refractory	26(70.3)
Number of previous lines treatment (median, range)	2(1–6)

The median number of previous lines of treatment was 2 (range 1–6). Thirty-five patients received HD-MTX-based chemotherapy as a first-line treatment. Four patients received WBRT (1 as induction, 1 as consolidation, and 2 as salvage treatment). One patient received ASCT as consolidation before relapse. Six patients used ibrutinib as a salvage treatment. The median time from onset to first progression of the disease was 7 months (range 0–96).

There were 28 patients who experienced disease relapse. Twenty-one patients relapsed in a location distinct from the initial disease. In this subgroup, 23 had disease involving deep brain lesions, while this number was 14 at the initial disease. At relapse, 4, 3, and 2 patients had intraocular, CSF, and spinal cord involvement, respectively (Table 2).

**Table 2:** Characteristics of patients received Ore-MTD or Ore-MIED.

Characteristics	Ore-MTD (*n* = 11, %)	Ore-MIED (*n* = 26, %)	*P* (Fisher)
Relapse	5(45.5)	6(23.1)	0.244
Refractory	6(54.5)	20(76.9)	0.244
Age >60	8(72.7)	2(7.7)	<0.001
SCNSL	2(18.2)	1(3.8)	0.205
previous treatment>3 lines	1(9.1)	7(26.9)	0.391
Previous treatment of BTKi	1(9.1)	5(19.2)	0.646
WBRT	0(0)	4(15.4)	0.296
Involved of deep lesions	11(100)	19(73.1)	0.080
CSF	2(18.2)	1(3.8)	0.205
Spinal cord	2(18.2)	1(3.8)	0.205
Bcl-2 c-myc double expression	7(63.6%)	11(42.3%)	0.295

### Treatment and response

Among 37 participants, the median number of treatment cycles was 3 cycles (range 1–6), 11 (29.7%) received Ore-MTD, and 26 (70.3%) received the Ore-MIED regimen. There were 8 (72.7%) and 2 (7.7%) patients aged more than 60 years in the Ore-MTD and Ore-MIED groups, respectively (*p* < 0.001). In the Ore-MTD regimen group, 5 (45.5%) had relapsed disease, and 6 (54.5%) had refractory disease, while in the Ore-MIED regimen group, 6 (23.1%) and 20 (76.9%) had relapsed and refractory disease (*p* = 0.244), respectively.

**Figure 1:**
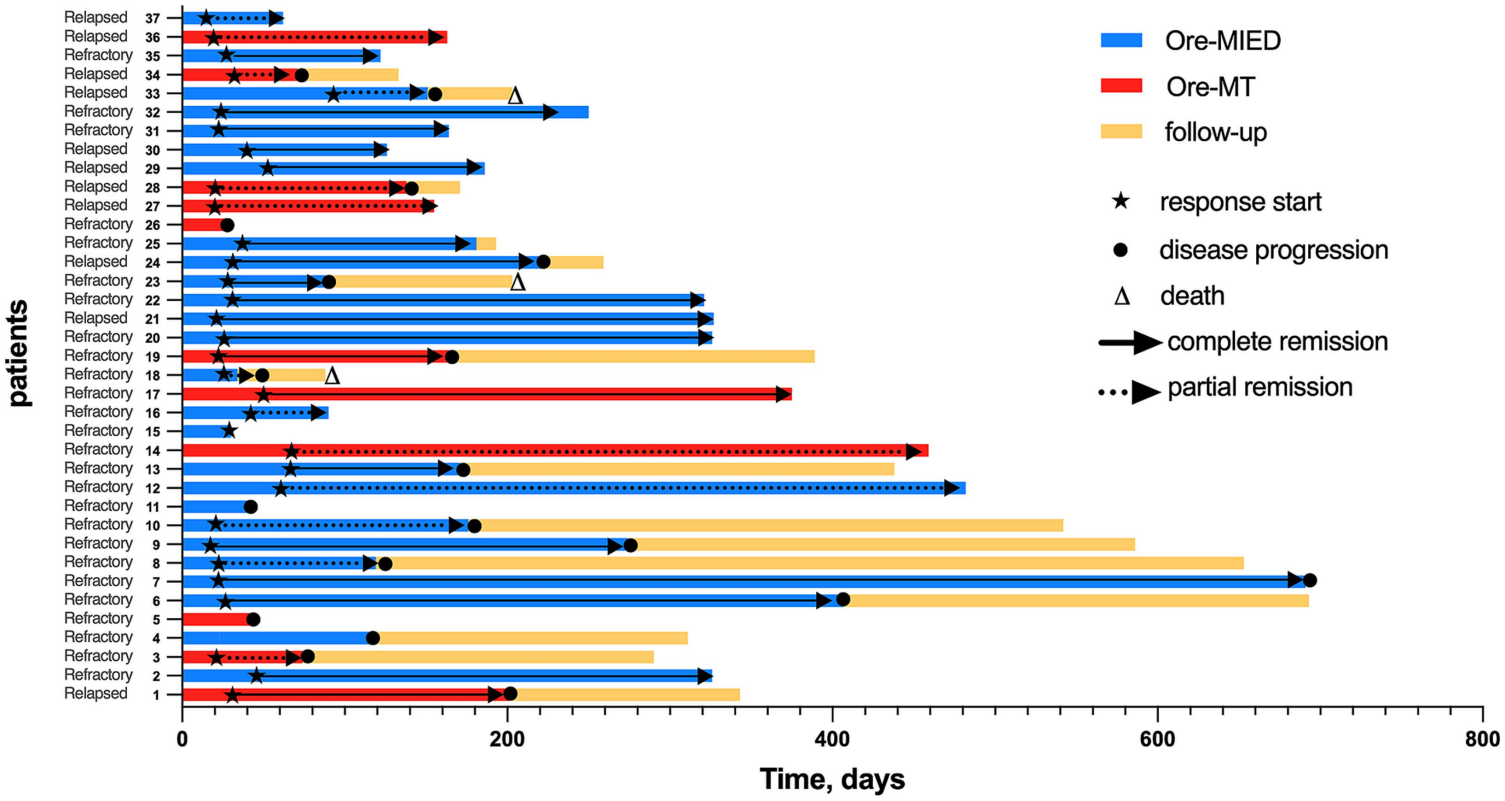
Swim lane plot of response and duration for all patients in the study (*n* = 37).

The CR and PR were observed in 19 (51.4%) and 14 (37.8%) patients, respectively, with an ORR of 89.2%. In patients who responded to treatment, the best responses were observed at a median of 3 cycles (range 1–6) (Figure 1). Among the 37 patients, 28 (75.7%) received fewer than 6 cycles of treatment, while 9 (24.3%) completed all 6 cycles. The reasons for discontinuation in the 28 patients who did not complete 6 cycles were as follows: 19 patients (67.9%) switched to alternative therapies due to failure to achieve complete remission (CR), 7 patients (25.0%) voluntarily discontinued induction therapy after achieving CR, and 2 patients (7.1%) withdrew due to renal insufficiency.

**Figure 2:**
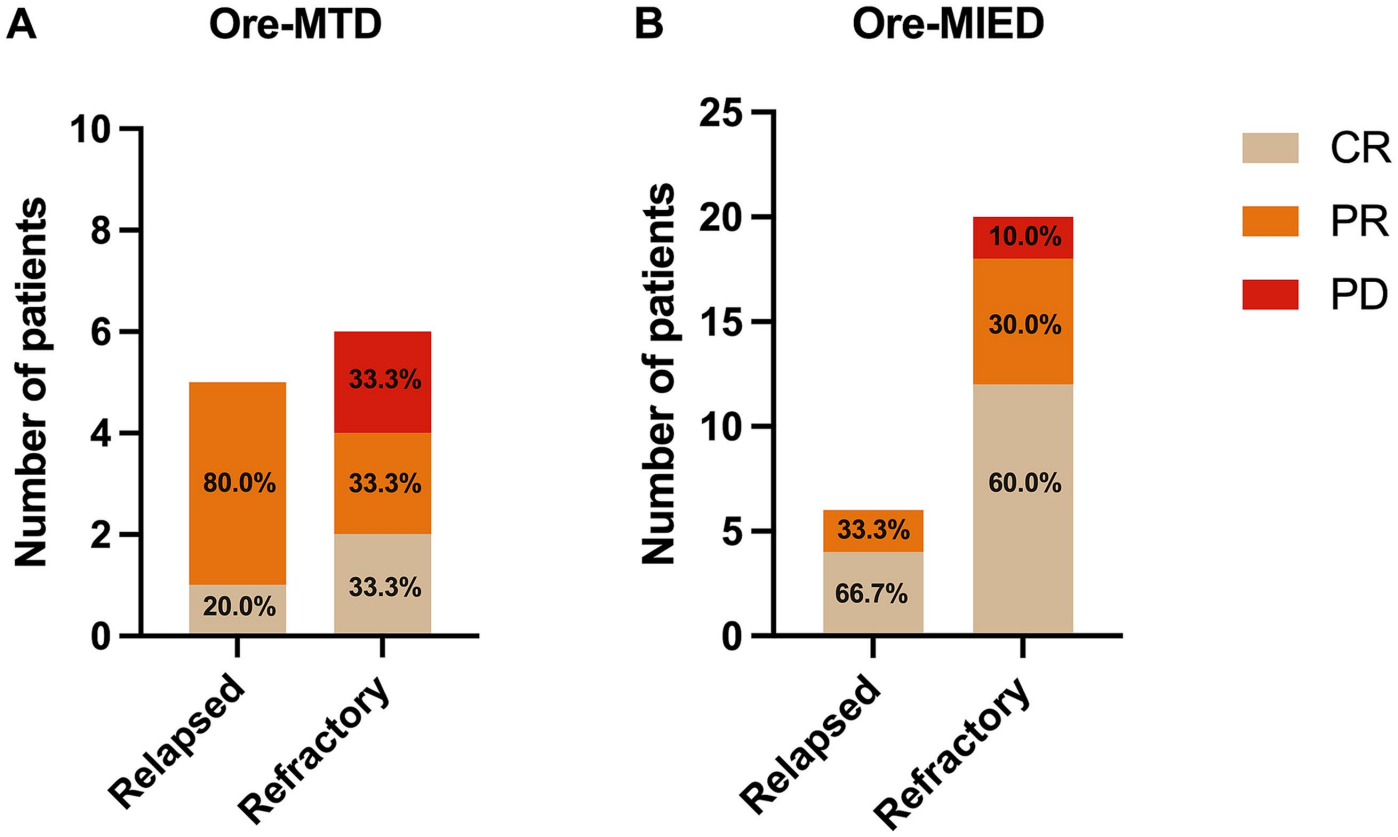
Treatment and responses among the relapse and refractory groups. Panel **(A)** in the Ore-MTD group, there were 5 patients with relapsed disease, of whom 1 achieved CR and 4 had PR; in the Ore-MTD group with refractory disease, there were 2 patients in the CR, PR, and PD groups; panel **(B)** in the Ore-MIED group, there were 6 patients with relapsed disease, and 4 and 2 patients achieved CR and PR, respectively; in the Ore-MIED group, there were 12, 6 and 2 patients who had CR, PR and PD among refractory disease patients, respectively.

In the Ore-MTD regimen group, three (27.3%) achieved CR, while 6 (54.5%) achieved PR resulting in an ORR of 81.8%. For the Ore-MIED regimen group, the ORR was 92.3%, with 16 (61.5%) patients achieving CR and 8 (30.8%) achieving PR. There was no statistically significant difference in ORR between the Ore-MT and the Ore-MIED regimens (*p* = 0.567) (Figure 2). Despite treatment regimen, patients who completed >3 cycles (*n* = 18) exhibited a higher CR rate (83.3% vs. 21.05% in those receiving ≤3 cycles, *p* = 0.0002).

Of six patients who had received first-generation BTKi ibrutinib, 1 received Ore-MT and experienced progressive disease after treatment. Five patients received Ore-MIED, 4 of whom achieved CR, and 1 had progression disease (PD).

### Survival

**Figure 3:**
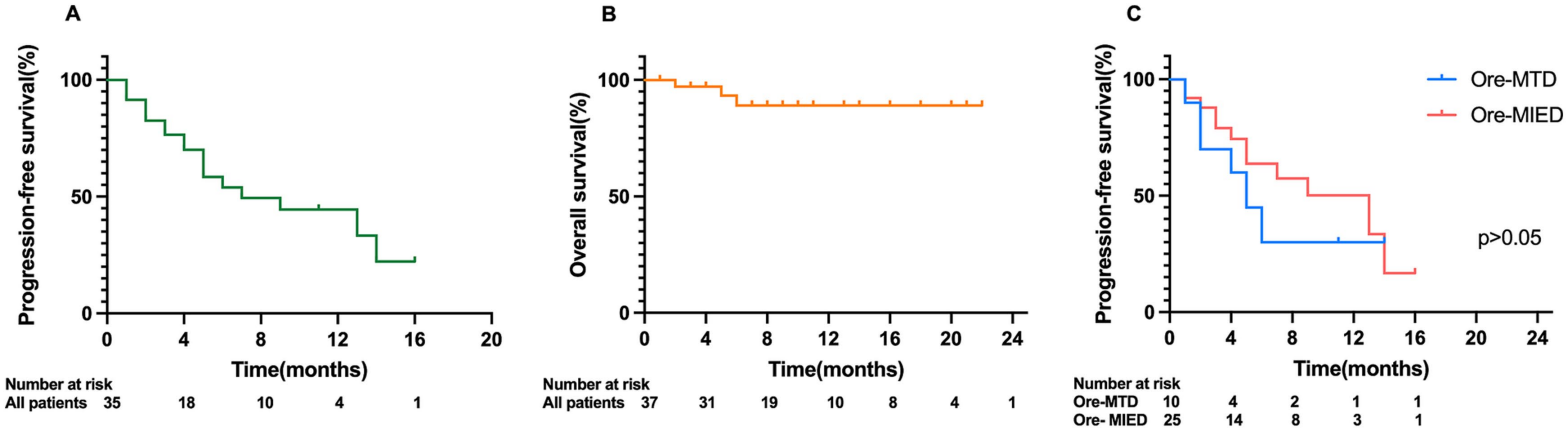
Kaplan–Meier analysis of PFS and OS in CNSL patients. **(A)** Kaplan–Meier analysis of PFS of all patients; **(B)** Kaplan–Meier analysis of OS of all patients; **(C)** comparison of PFS between the Ore-MTD and Ore-MIED groups.

The median follow-up period in all 37 patients was 8.0 months (range 1.0–23.0 months). The median time to treatment response (TTR) was 0.55 ± 0.56 months. The median PFS was 7.0 months (95% CI, 1.525–12.475). There was no significant difference in median PFS between different treatment regimens. The median PFS in the Ore-MTD and Ore-MIED groups was 5.0 months (95% CI, 0.778–9.222) and 13.0 (95% CI, 5.235–20.765), respectively (*p* = 0.293) (Figure 3). The median OS among all cases was not reached in this cohort. Univariate analysis indicated that CSF relapse and spinal cord relapse were associated with shorter PFS (*p* = 0.011 and *p* = 0.019).

We included age, disease status (SCNSL or PCNSL/relapse or refractory), previous treatment (treatment lines exceeded 3/previous BTKi/previous WBRT), completing>3 cycles of treatment, and location of disease (deep lesion/CSF/intraocular/spinal cord) in univariate and multivariate analyses with PFS. Multivariate analysis identified CSF relapse as an independent risk factor for PFS (*p* = 0.018) (Supplementary Data 2). There were not enough events to perform univariate and multivariate analyses for OS using Cox proportional hazards regression models.

### Toxicity

The overall toxicities are shown in Table 3 (toxicities in subgroups are shown in Supplementary Data 3). Hematological toxicity was common; 5 patients experienced grade 4 hematological toxicities, 4 of which were from the Ore-MIED group, while there was only one grade 4 toxicity in the Ore-MTD group (thrombocytopenia). All other toxicities were limited to grades 1–2, and grade 1–2 liver toxicity and nephrotoxicity were detected in 48.6 and 35.1% of patients, respectively. Grade 1–2 purpura was observed in 13.5% of patients, among which 1 patient received an orelabrutinib dose of 50 mg. We did not observe fungal infection or cardiac arrhythmia in this cohort, and there was no treatment-related death.

**Table 3:** Overall toxicities of patients received Ore-MTD or Ore-MIED (*n* = 37).

Toxicity	Grade 1–2 (*n*, %)	Grade 3 (*n*, %)	Grade 4 (*n*, %)
Hematological toxicity			
Leukopenia	9(24.3)	7(18.9)	1(2.7)
Neutropenia	9(24.3)	5(13.5)	2(5.4)
Anemia	27(73.0)	3(8.1)	0
Thrombocytopenia	8(21.6)	3(8.1)	2(5.4)
Infection	2(5.4)	0	0
Purpura	5(13.5)	0	0
Liver toxicity			
Aminotransferases elevated	13(35.1)	0	0
Bilirubin elevated	18(48.6)	0	0
Nephrotoxicity			
Creatinine elevated	13(35.1)	0	0
Gastrointestinal reaction			
Mucositis	0	0	0
Inappetence	3(8.1)	0	0
Anaphylaxis	0	0	0

## Discussion

In this single-center retrospective study, we described the characteristics, treatment response, and survival of 37 rrCNSL patients using orelabrutinib-based regimens. Ore-MTD and Ore-MIED revealed favorable therapeutic activity in rrCNSL.

Currently, there is no established standard regimen for rrPCNSL. According to the National Comprehensive Cancer Network (NCCN) guidelines, HD-MTX rechallenge is recommended in rrPCNSL when the duration of first response exceeded 12 months (14). Repeat induction with HD-MTX seems to be more effective in managing relapsed PCNSL. In a retrospective study involving 22 patients with relPCNSL, the use of HD-MTX as a single agent for repeat induction resulted in a high ORR of 91% and a CR rate of 73%. Another study, including 39 relapsed PCNSL patients, employed a regimen that incorporated HD-MTX and achieved an ORR of 85% with a CR rate of 74% (2). It’s worth noting that different drug combinations, particularly when combined with BTK inhibitors, may yield varying treatment outcomes. Therefore, in this research, we did not take the duration of remission achieved after initial therapy to be an absolute criterion for selecting a treatment regimen and included HD-MTX in both groups.

Recurrent mutations in the JAK–STAT, NF-κB, and B-cell receptor (BCR) pathways, including *CD79B, MYD88*, and *CDKN2A* deletions, have been identified as the defining characteristics of primary central nervous system lymphomas (15–17). The introduction of Bruton tyrosine kinase inhibitors has significantly improved the clinical response in certain cases of relapsed or refractory PCNSL by targeting the BCR signaling pathway (11). BTKis were also reported to be capable of modulating B-cell functions and attenuating microglial activities (18). Studies report variable efficacy for ibrutinib combinations in CNSL, DA-TEDDi-R resulted in a complete response rate of 86% (19), though with toxicity risks like aspergillosis, while temozolomide monotherapy shows lower response rates (ORR: 31%) (6, 13, 20, 21). Real-world studies have indicated that approximately 19–49% of patients discontinue ibrutinib, with intolerance being the most common reason for discontinuation rather than disease progression (22). BTKi resistance was found to be induced by CD79B overexpression through AKT/MAPK pathways in DLBCL (23).

Next-generation BTKis have been developed to reduce toxicity and side effects while improving tolerability and decreasing the risk of bleeding and cardiac arrhythmias through more selective kinase inhibition. Orelabrutinib, as a novel irreversible covalent BTK inhibitor features optimized structure with improved kinase selectivity and reduced off-target effects, demonstrating over 90% inhibition only on BTK at 1 μmol/L concentration among 456 tested kinases (24, 25). Its unique single-ring core and minimized steric hindrance enhance target specificity, leading to better efficacy and safety (26, 27). Chinese patients with relapsed and refractory mantle cell lymphoma have demonstrated sustained efficacy and well tolerance with orelabrutinib in two studies (28, 29). Also, orelabrutinib exhibits a high ability to penetrate the blood–brain barrier, which results in a high cerebrospinal fluid (CSF)/plasma ratio (30). Emerging evidence in retrospective studies and an ongoing Phase II trial (ICP-CL-00106) supports its use in CNSL (30–33) (detailed summary of orelabrutinib-Based chemotherapy regimens for CNSL see in Supplementary Data 4).

Against the backdrop of emerging new drugs, finding treatment options for rrPCNSL has become increasingly challenging and requires highly individualized approaches. The choice of suitable treatment for rrCNSL largely depends on factors such as age, efficacy and toxicity of previous treatment. Additionally, many rrPCNSL patients may not be suitable candidates for further intensive therapy due to advanced age at relapse, compromised performance status, neurocognitive dysfunction, poor physical fitness, or comorbid conditions (5). In our study, we considered the varying baseline conditions of patients and offered two treatment options. Ore-MTD provided an alternative for elderly and frail patients who could not tolerate intensive treatment, whereas Ore-MIED represented a relatively intense regimen. Although there was no significant difference in the overall response rate (ORR) or median PFS between Ore-MTD and Ore-MIED, patients receiving Ore-MIED exhibited a higher ORR and longer PFS, suggesting that better and longer responses could be achieved with an aggressive regimen.

Our team reported the efficacy of the I-MIDD regimen (ibrutinib/methotrexate/ifosfamide/liposomal doxorubicin/methylprednisolone) in 18 rrPCNSL patients, with a ORR of 83.3% and a median PFS of 6 (1–27) months (34). In a small retrospective study, 15 rrPCNSL patients achieved an ORR of 86.7% with rituximab, HD-MTX, temozolomide, orelabrutinib, and lenalidomide (31). Another retrospective study included both rrPCNSL and treatment-naive PCNSL patients. Of the 15 rrCNSL patients, the ORR was 60.00% using orelabrutinib-containing regimens (30). Despite the inclusion of patients previously treated with ASCT, WBRT, and first-generation BTKis, the survival and response to treatment were consistent with previous studies using orelabrutinib and comparable to other BTKi-containing regimens. Regarding safety, in our study, we did not observe any cases of atrial fibrillation or invasive fungal infections, which are commonly associated adverse events with BTKis. This observation aligns with prior reports emphasizing the remarkable target specificity and minimal off-target response of orelabrutinib (30).

The identification of prognostic factors in rrPCNSL remains somewhat imprecise. Existing prognostic models are primarily based on clinical characteristics at baseline, without any accounting for dynamic factors. In our study, we identified CSF relapse as an independent risk factor for PFS, which indicates that rrCNSL patients with CSF relapse may need intense treatment (35, 36). Furthermore, rrPCNSL tends to recur in distinct anatomical locations from the primary tumor. In our study, 21 out of 28 patients experienced relapses in a location different from the initial disease, with an increased proportion of deep lesions. This suggests a more aggressive nature of rrCNSL, as deep lesion involvement has been identified as an inferior prognostic factor in newly diagnosed PCNSL (35). Notably, patients who completed >3 cycles exhibited a higher CR rate However, univariate analysis revealed no significant difference in median PFS between patients completing >3 cycles and those receiving ≤3 cycles. This suggests that while extended treatment duration correlates with deeper responses, it may not independently prolong PFS in this cohort, potentially due to confounding factors such as heterogeneous salvage therapies post-discontinuation or intrinsic disease aggressiveness. Larger sample studies are needed to validate above conclusions.

There are a few limitations in this study. Due to the limited sample size, conducting multivariate analysis was constrained in this study. Furthermore, the median follow-up time in this study is relatively short. In future studies, scholars should aim to expand sample size and to extend the follow-up period to further explore the prognosis of rrCNSL. Additionally, the lack of genome sequencing and MYC rearrangement on tumor samples is another limitation.

## Conclusion

Our findings demonstrate that Ore-MTD and Ore-MIED are both tolerable and clinically active in rrCNSL, including patients with prior exposure to multiple lines of therapy, including WBRT and the first generation BTKi. These results position orelabrutinib as a promising therapeutic backbone for rrCNSL, particularly in populations with limited salvage options.

## Data Availability

The raw data supporting the conclusions of this article will be made available by the authors, without undue reservation.

## Glossary

rrCNSL: Relapsed or refractory central nervous system lymphoma
BTKi: Bruton’s tyrosine kinase inhibitor
Ore-MIED: orelabrutinib, high-dose methotrexate, ifosfamide, etoposide, and dexamethasone
Ore-MTD: orelabrutinib, high-dose methotrexate, temozolomide and dexamethasone
PCNSL: primary central nervous system lymphoma
SCNSL: secondary central nervous system lymphoma
HD-MTX: high-dose methotrexate
WBRT: whole brain radiotherapy
TIER: thiotepa/rituximab/ifosfamide/etoposide
ASCT: autologous hematopoietic stem cell transplantation
ORR: overall response rate
PFS: progression-free survival
OS: overall survival
CSF: cerebrospinal fluid
DLBCL: diffuse large B-cell lymphoma
Rel: Relapse
Ref: Refractory
CR: complete remission
CRu: unconfirmed complete response
PR: partial response
PD: progression disease
GCB: germinal center B cells
IELSG: International Extranodal Lymphoma Study Group
PET-CT: positron emission tomography scan
CNS: central nervous system
NCCN: National Comprehensive Cancer Network
BCR: B-cell receptor

## References

[1] Plotkin SR Betensky RA Hochberg FH Grossman SA Lesser GJ Nabors LB. Treatment of relapsed central nervous system lymphoma with high-dose methotrexate. Clin Cancer Res. (2004) 10:5643–6. doi: 10.1158/1078-0432.CCR-04-0159, PMID: 15355887

[2] Pentsova E Deangelis LM Omuro A. Methotrexate re-challenge for recurrent primary central nervous system lymphoma. J Neurooncol. (2014) 117:161–5. doi: 10.1007/s11060-014-1370-0, PMID: 24481997 PMC5256683

[3] Sun X Lv L Wu Y Cui Q Sun S Ji N. Challenges in the management of primary central nervous system lymphoma. Crit Rev Oncol Hematol. (2023) 188:104042. doi: 10.1016/j.critrevonc.2023.104042, PMID: 37277008

[4] Langner-Lemercier S Houillier C Soussain C Ghesquières H Chinot O Taillandier L. Primary CNS lymphoma at first relapse/progression: characteristics, management, and outcome of 256 patients from the French LOC network. Neuro Oncol. (2016) 18:1297–303. doi: 10.1093/neuonc/now033, PMID: 26951382 PMC4998995

[5] Ambady P Doolittle ND Fox CP. Relapsed and refractory primary CNS lymphoma: treatment approaches in routine practice. Ann Lymphoma. (2021) 5:23. doi: 10.21037/aol-21-20, PMID: 35253010 PMC7612457

[6] Reni M Zaja F Mason W Perry J Mazza E Spina M. Temozolomide as salvage treatment in primary brain lymphomas. Br J Cancer. (2007) 96:864–7. doi: 10.1038/sj.bjc.6603660, PMID: 17325700 PMC2360092

[7] Constine LS Yahalom J Ng AK Hodgson DC Wirth A Milgrom SA. The role of radiation therapy in patients with relapsed or refractory Hodgkin Lymphoma: guidelines from the international lymphoma radiation oncology group. Int J Radiat Oncol Biol Phys. (2018) 100:1100–18. doi: 10.1016/j.ijrobp.2018.01.011, PMID: 29722655

[8] Houillier C Choquet S Touitou V Martin-Duverneuil N Navarro S Mokhtari K. Lenalidomide monotherapy as salvage treatment for recurrent primary CNS lymphoma. Neurology. (2015) 84:325–6. doi: 10.1212/WNL.0000000000001158, PMID: 25527263

[9] Fox CP Ali AS McIlroy G Thust S Martinez-Calle N Jackson AE. A phase 1/2 study of thiotepa-based immunochemotherapy in relapsed/refractory primary CNS lymphoma: the TIER trial. Blood Adv. (2021) 5:4073–82. doi: 10.1182/bloodadvances.2021004779, PMID: 34464973 PMC8945638

[10] Liu J Guo J Sun X Liu Y Gao C. Efficacy and safety of autologous stem-cell transplantation as part of first-line treatment for newly diagnosed primary central nervous system lymphoma: a systematic review and meta-analysis. Front Oncol. (2021) 11:799721. doi: 10.3389/fonc.2021.799721, PMID: 35096600 PMC8790123

[11] Lv L Sun X Wu Y Cui Q Chen Y Liu Y. Efficacy and safety of ibrutinib in central nervous system lymphoma: a PRISMA-compliant single-arm meta-analysis. Front Oncol. (2021) 11:707285. doi: 10.3389/fonc.2021.707285, PMID: 34277452 PMC8280788

[12] Grommes C Pastore A Palaskas N Tang SS Campos C Schartz D. Ibrutinib unmasks critical role of Bruton tyrosine kinase in primary CNS lymphoma. Cancer Discov. (2017) 7:1018–29. doi: 10.1158/2159-8290.CD-17-0613, PMID: 28619981 PMC5581705

[13] Grommes C Tang SS Wolfe J Kaley TJ Daras M Pentsova EI. Phase 1b trial of an ibrutinib-based combination therapy in recurrent/refractory CNS lymphoma. Blood. (2019) 133:436–45. doi: 10.1182/blood-2018-09-875732, PMID: 30567753 PMC6356986

[14] Chaiyachati KH Krause D Sugalski J Graboyes EM Shulman LN. A survey of the national comprehensive cancer network on approaches toward addressing patients' transportation insecurity. J Natl Compr Canc Netw. (2023) 21:21–6. doi: 10.6004/jnccn.2022.7073, PMID: 36634609 PMC9888481

[15] Radke J Ishaque N Koll R Gu Z Schumann E Sieverling L. The genomic and transcriptional landscape of primary central nervous system lymphoma. Nat Commun. (2022) 13:2558. doi: 10.1038/s41467-022-30050-y, PMID: 35538064 PMC9091224

[16] Nakamura T Tateishi K Niwa T Matsushita Y Tamura K Kinoshita M. Recurrent mutations of CD79B and MYD88 are the hallmark of primary central nervous system lymphomas. Neuropathol Appl Neurobiol. (2016) 42:279–90. doi: 10.1111/nan.12259, PMID: 26111727

[17] Severson EA Haberberger J Hemmerich A Huang R Edgerly C Schiavone K. Genomic profiling reveals differences in primary central nervous system lymphoma and large B-cell lymphoma, with subtyping suggesting sensitivity to BTK inhibition. Oncologist. (2023) 28:e26–26e35. doi: 10.1093/oncolo/oyac190, PMID: 36342081 PMC9847534

[18] Cao T Wang Z Zhu X. The immunomodulatory functions of BTK inhibition in the central nervous system. J Inflamm Res. (2022) 15:6427–38. doi: 10.2147/JIR.S389958, PMID: 36452053 PMC9704002

[19] Lionakis MS Dunleavy K Roschewski M Widemann BC Butman JA Schmitz R. Inhibition of B cell receptor signaling by ibrutinib in primary CNS lymphoma. Cancer Cell. (2017) 31:833–43.e5. doi: 10.1016/j.ccell.2017.04.012, PMID: 28552327 PMC5571650

[20] Renaud L Bossard JB Carpentier B Terriou L Cambier N Chanteau G. Treatment with temozolomide and ibrutinib in recurrent/refractory primary (PCNSL) and secondary CNS lymphoma (SCNSL). Eur J Haematol. (2021) 107:370–3. doi: 10.1111/ejh.13667, PMID: 34018260

[21] Makino K Nakamura H Hide T Kuratsu J. Salvage treatment with temozolomide in refractory or relapsed primary central nervous system lymphoma and assessment of the MGMT status. J Neurooncol. (2012) 106:155–60. doi: 10.1007/s11060-011-0652-z, PMID: 21720808

[22] Mato AR Nabhan C Thompson MC Lamanna N Brander DM Hill B. Toxicities and outcomes of 616 ibrutinib-treated patients in the United States: a real-world analysis. Haematologica. (2018) 103:874–9. doi: 10.3324/haematol.2017.182907, PMID: 29419429 PMC5927982

[23] Kim JH Kim WS Ryu K Kim SJ Park C. CD79B limits response of diffuse large B cell lymphoma to ibrutinib. Leuk Lymphoma. (2016) 57:1413–22. doi: 10.3109/10428194.2015.1113276, PMID: 26699656

[24] Dhillon S. Orelabrutinib: first approval. Drugs. (2021) 81:503–7. doi: 10.1007/s40265-021-01482-5, PMID: 33704654

[25] Kaptein A de Bruin G Emmelot-van Hoek M van de Kar B de Jong A Gulrajani M. Potency and selectivity of BTK inhibitors in clinical development for B-cell malignancies. Blood. (2018) 132:1871. doi: 10.1182/blood-2018-99-10997330082493

[26] Xu W Song Y Li Z Yang S Liu L Hu Y. Safety, tolerability and efficacy of orelabrutinib, once a day, to treat Chinese patients with relapsed or refractory chronic lymphocytic leukemia/small cell leukemia. Blood. (2019) 134:4319–9. doi: 10.1182/blood-2019-123331

[27] Yu H Wang X Li J Ye Y Wang D Fang W. Addition of BTK inhibitor orelabrutinib to rituximab improved anti-tumor effects in B cell lymphoma. Mol Ther Oncolytics. (2021) 21:158–70. doi: 10.1016/j.omto.2021.03.015, PMID: 33981831 PMC8082047

[28] Deng L Li Z Zhang H Huang H Hu J Liu L. Orelabrutinib for the treatment of relapsed or refractory marginal zone lymphoma: a phase 2, multicenter, open-label study. Am J Hematol. (2023) 98:1742–50. doi: 10.1002/ajh.27064, PMID: 37647123

[29] Deng LJ Zhou KS Liu LH Zhang MZ Li ZM Ji CY. Orelabrutinib for the treatment of relapsed or refractory MCL: a phase 1/2, open-label, multicenter, single-arm study. Blood Adv. (2023) 7:4349–57. doi: 10.1182/bloodadvances.2022009168, PMID: 37078706 PMC10432605

[30] Wu JJ Wang WH Dong M Ma SS Zhang XD Zhu LN. Orelabrutinib-bruton tyrosine kinase inhibitor-based regimens in the treatment of central nervous system lymphoma: a retrospective study. Invest New Drugs. (2022) 40:650–9. doi: 10.1007/s10637-022-01219-5, PMID: 35137332

[31] Yang C Cui Y Ren X Li M Yu K Shen S. Orelabrutinib combined with lenalidomide and immunochemotherapy for relapsed/refractory primary central nervous system lymphoma: a retrospective analysis of case series. Front Oncol. (2022) 12:901797. doi: 10.3389/fonc.2022.901797, PMID: 35785180 PMC9243261

[32] Chen T Liu Y Wang Y Chang Q Wu J Wang Z. Evidence-based expert consensus on the management of primary central nervous system lymphoma in China. J Hematol Oncol. (2022) 15:136. doi: 10.1186/s13045-022-01356-7, PMID: 36176002 PMC9524012

[33] Li Y Li Y Zeng R He Y Liang L Ou L. High-dose methotrexate, thiotepa, orelabrutinib combined with or without rituximab in primary or secondary central nervous system diffuse large B-cell lymphoma: a single-center retrospective analysis. J Cancer. (2023) 14:3182–90. doi: 10.7150/jca.85756, PMID: 37928429 PMC10622989

[34] Yuedan CSX Bai XCQ Liu Y. Preliminary exploration of ibrutinib combined with methotrexate, ifosfamide, liposomal doxorubicin and methylprednisolone in the treatment of relapsed/refractory primary CNS lymphoma. Blood. (2020) 136:13–4. doi: 10.1182/blood-2020-141756

[35] Ferreri AJ Blay JY Reni M Pasini F Spina M Ambrosetti A. Prognostic scoring system for primary CNS lymphomas: the International Extranodal Lymphoma Study Group experience. J Clin Oncol. (2003) 21:266–72. doi: 10.1200/JCO.2003.09.139, PMID: 12525518

[36] Abrey LE Ben-Porat L Panageas KS Yahalom J Berkey B Curran W. Primary central nervous system lymphoma: the Memorial Sloan-Kettering Cancer Center prognostic model. J Clin Oncol. (2006) 24:5711–5. doi: 10.1200/JCO.2006.08.2941, PMID: 17116938

