# Supplementary material for: Efficacy of individualized orelabrutinib-based regimens in relapsed or refractory central nervous system lymphoma
Source: Front Neurol. 2025 Jun 5;16:1570224. doi: 10.3389/fneur.2025.1570224 (PMC12176547; doi:10.3389/fneur.2025.1570224)
Supplement: Supplementary file 1 [file Data_Sheet_1.docx]

**Supplementary data 1**

1. Ore-MTD

Oral Orelabrutinib 150mg once a day

Day 1：Methotrexate 3.5g/m^2^ IV within 3.5 hours.

Day 2： Leucovorin rescue every 6 hours until the methotrexate < 0.10 μmol/L.

Day 2-6: Temozolomide 100-150mg/m2

Day 1-3: Dexamethasone 10mg IV.

1. Ore-MIED

Oral Orelabrutinib 150mg once a day

Day 1：Methotrexate 3.5g/m^2^ IV within 3.5 hours.

Day 2：Ifosfamide 1.5 g/m^2^ IV ; Leucovorin rescue every 6 hours until the methotrexate < 0.10 μmol/L.

Day 3: Etoposide 100mg/m^2^ IV.

Day 1-3: Dexamethasone 10mg IV.

The dose of HD-MTX and Temozolomide depended on the patient age and Eastern Cooperative Oncology Group (ECOG) performance status. Due to age and ECOG performance status, temozolomide was administered at a dosage of 100 mg/m² for three patients, while other patients received temozolomide at a dosage of 150 mg/m². Methotrexate was initiated at 3.5 mg/m² for all patients. For those who developed Grade 1 or higher renal impairment following treatment, the methotrexate dosage was reduced to 3.0 g/m².Ore-MTD or Ore-MIED was administered every 3 weeks for 3-6 cycles.

**Supplementary data 2**

Univariate and multivariate analyses of PFS for patients (Cox test)

|  | PFS | | |  | PFS | | |
| --- | --- | --- | --- | --- | --- | --- | --- |
|  | univariate analysis | | |  | multivariate analysis | | |
|  | HR | 95%CI | p |  | HR | 95%CI | p |
| Age>60 | 1.950 | 0.707-5.377 | 0.197 |  |  |  |  |
| Refractory disease | 0.908 | 0.326-2.706 | 0.908 |  |  |  |  |
| Previous WBRT | 2.516 | 0.696-9.096 | 0.159 |  |  |  |  |
| Previous lines of treatment>3 | 0.550 | 0.177-1.710 | 0.302 |  |  |  |  |
| Previous BTKi treatment | 0.512 | 0.142-1.849 | 0.307 |  |  |  |  |
| Spinal cord relapse | 5.024 | 1.305-19.342 | 0.019 |  | 7.997 | 0.900-71.068 | 0.062 |
| Deep lesion involved in relapse | 1.111 | 0.359-3.434 | 0.855 |  |  |  |  |
| Intraocular relapse | 0.533 | 0.122-2.336 | 0.404 |  |  |  |  |
| BCL-2, MYC double expression | 1.311 | 0.529-3.251 | 0.558 |  |  |  |  |
| CSF relapse | 5.746 | 1.501-21.996 | 0.011 |  | 16.557 | 1.625-168.732 | 0.018 |
| SCNSL | 3.861 | 0.828-18.008 | 0.086 |  | 0.039 | 0.001-1.234 | 0.066 |
| Completing>3cycles of treatment | 1.132 | 0.442-2.897 | 0.797 |  |  |  |  |

**Supplementary data 3**

Toxicities in Ore-MTD regimen group(n=11).

| Toxicity | Grade 1-2 | Grade 3 | Grade 4 |
| --- | --- | --- | --- |
| Hematological toxicity |  |  |  |
| Leukopenia | 2(18.2) | 1(9.1) | 0 |
| Neutropenia | 2(18.2) | 1(9.1) | 0 |
| Anemia | 6(54.5) | 1(9.1) | 0 |
| Thrombocytopenia | 0 | 1(9.1) | 1(9.1) |
| Infection | 0 | 0 | 0 |
| Purpura | 2(18.2) | 0 | 0 |
| Liver toxicity |  |  |  |
| Aminotransferases elevated | 3(27.3) | 0 | 0 |
| Bilirubin elevated | 6(54.5) | 0 | 0 |
| Nephrotoxicity |  |  |  |
| Creatinine elevated | 5 (45.5) | 0 | 0 |
| Gastrointestinal reaction |  |  |  |
| Mucositis | 0 | 0 | 0 |
| Inappetence | 3(27.3) | 0 | 0 |
| Anaphylaxis | 0 | 0 | 0 |

Toxicity in Ore-MIED regimen group(n=26).

| Toxicity | Grade 1-2 | Grade 3 | Grade 4 |
| --- | --- | --- | --- |
| Hematological toxicity |  |  |  |
| Leukopenia | 7(26.9) | 6(23.1) | 1(3.8) |
| Neutropenia | 7(26.7) | 4(15.4) | 2(7.7) |
| Anemia | 21(80.8) | 2(7.7) | 0 |
| Thrombocytopenia | 8(20.8) | 2(7.7) | 1(3.8) |
| Infection | 2(7.7) | 0 | 0 |
| Purpura | 3(11.5) | 0 | 0 |
| Liver toxicity |  |  |  |
| Aminotransferases elevated | 10(38.5) | 0 | 0 |
| Bilirubin elevated | 12(46.2) | 0 | 0 |
| Nephrotoxicity |  |  |  |
| Creatinine elevated | 8(30.8) | 0 | 0 |
| Gastrointestinal reaction |  |  |  |
| Mucositis | 0 | 0 | 0 |
| Inappetence | 0 | 0 | 0 |
| Anaphylaxis | 0 | 0 | 0 |

**Supplementary data 4**

**Research Summary of Orelabrutinib-Based Chemotherapy Regimens for Central Nervous System Lymphoma**

| Study | Regimen | Patient Population | Sample Size | ORR (%) | Median PFS (months) | Median OS  (months) | Grade 3-4  AEs (%) |
| --- | --- | --- | --- | --- | --- | --- | --- |
| Li et al.  (2023) | HD-MTX + Thiotepa + Orelabrutinib ± Rituximab | PCNSL/SCNSL (Newly Diagnosed/Relapsed/Refractory) | 14  (R/R=12) | 91.8 in R/R subgroup(8CR or CRu) | 12 months PFS rate 60% | 12 month OS rate 70% | 35.7 |
| Wu et al. (2022) | Orelabrutinib ± Immunotherapy/Chemotherapy/Radiotherapy | PCNSL/SCNSL (Newly Diagnosed/Relapsed/Refractory) | 23  (R/R=15) | 60.0 in R/R subgroup(4CR,5PR) | 6 month PFS rate 92.30% | 6 month OS rate 67.70% | 26.1 |
| Yang et al. (2022) | Orelabrutinib + Lenalidomide + Rituximab + HD-MTX + Temozolomide (RMT) | Relapsed/Refractory PCNSL | 15 | 86.7 | 9.8 | Not reached | 40 (Fatigue) |
